# Cardio-Cerebrovascular Disease is Associated With Severity and Mortality of COVID-19: A Systematic Review and Meta-Analysis

**DOI:** 10.1177/1099800420951984

**Published:** 2020-08-27

**Authors:** Jia-Ning Yu, Bing-Bing Wu, Jie Yang, Xiao-Ling Lei, Wang-Qin Shen

**Affiliations:** 1 School of Medicine, 66479 Nantong University, Jiangsu, People’s Republic of China

**Keywords:** cardio-cerebrovascular disease, COVID-19, severity; mortality, meta-analysis

## Abstract

At present, COVID-19 is raging all over the world. Many comorbidities, such as diabetes mellitus (OR = 2.67, 95% CI = 1.91–3.74) and hypertension (OR = 2.3, 95% CI = 1.76–3.00), have been shown to worsen the patient’s condition. However, whether cardio-cerebrovascular disease will affect COVID-19 remains unclear. In this meta-analysis, we collected studies from PubMed, Wed of Science and CNKI (Chinese) to July 25, which reported COVID-19 patients with and without cardio-cerebrovascular disease as well as their severity and mortality. The random-effect model meta-analysis was used to analyze them and get overall odds ratios (OR) with 95% CIs. Funnel plots and the Begg’s and Egger’s test were used to assess publication bias. Thirty-one studies with 23,632 patients were finally included in the meta-analysis. The results showed an OR of 3.004 (95% CI = 2.097–4.303) for COVID-19 severity and an OR of 5.587 (95% CI = 2.810–11.112) for COVID-19 mortality. Compared with cardiovascular disease, the subgroup analysis indicated that cerebrovascular disease was more likely to increase the severity (OR = 3.400, 95% CI = 1.569–7.368) and mortality (OR = 23.477, 95% CI = 3.050–180.735) of COVID-19. Therefore, it can be inferred that cardio-cerebrovascular disease is associated with an increase in the risk of severe illness and death among COVID-19 patients. This meta-analysis showed that cardio-cerebrovascular disease has a significant relation with severe and death outcomes of COVID-19. Nurses should pay special attention to COVID-19 patients with the cardio-cerebrovascular disease.

Over the past 20 years, there were 2 coronaviruses of particular concern (Drosten et al., 2003): SARS-CoV in 2002 and MERS-CoV in 2012, which had nearly 50% mortality rates for certain populations. In December 2019, a new kind of coronavirus emerged in Wuhan, China (Chan et al., 2003; Drosten et al., 2003; Zhu et al., 2020). The pathogen was classified as a novel enveloped RNA β coronavirus. It has been named Severe Acute Respiratory Syndrome CoronaVirus (SARS-CoV-2), and results in significant morbidity and mortality (Hendren et al., 2020; Lu et al., 2020). As of July 27th, 2020, more than 15 million confirmed cases and 640,000 confirmed deaths have been officially documented worldwide. Patients’ clinical symptoms include fever, dry cough, fatigue, and organ dysfunction (Huang et al., 2020; Zhou et al., 2020). Because it has rapidly spread to 216 countries, areas or territories and it has transmitted throughout 6 continents, the Coronavirus Disease 2019 (COVID-19) was declared as a global pandemic by the World Health Organization (https://www.who.int/emergencies/diseases/novel-coronavirus-2019).

Recent studies have revealed that the specific symptoms and comorbidities may be related to COVID-19 severity and lead to a poor prognosis. Comorbidities such as diabetes mellitus (DM; OR = 2.67, 95% CI = 1.91–3.74) and chronic obstructive pulmonary disease (COPD; OR = 4.32, 95% CI = 2.34–8.30) are associated with higher disease severity in patients with COVID-19 (Y. Chen et al., 2020; Zhao et al., 2020). Meanwhile, some researches showed that cardio-cerebrovascular disease had a significant impact on risk for COVID-19 severity (OR = 0.164, 95% CI = 0.066–0.261; B. Li et al., 2020), and COVID-19 patients with cardio-cerebrovascular may have a higher risk of mortality (Yuan et al., 2020; Zhou et al., 2020). Nevertheless, few specific studies have focused on the impact of cardio-cerebrovascular disease on the prognosis in COVID-19 patients.

Therefore, we aimed to explore whether cardio-cerebrovascular disease would increase the risk of COVID-19 patients by a systematic review and meta-analysis of existing studies. We also aimed to discover the relationship between cardio-cerebrovascular disease and COVID-19 severity and mortality. We hope that our findings will shed a new light on better implementing nursing intervention, risk stratification and disease management of COVID-19 in patients with the cardio-cerebrovascular diseases.

## Methods

### Search Strategy

This study was conducted according to the Preferred Reporting Items for Systematic Reviews and Meta-Analyses (PRISMA) guidelines (Moher et al., 2009). We undertook a systematic search of PubMed (http://www.ncbi.nlm.nih.gov/pubmed), China National Knowledge Infrastructure Database, CNKI (https://www.cnki.net/), Web of Science, MedRxiv (http://www.medrxiv.org/) and BioRxiv (http://www.biorxiv.org) using the following search terms and relevant variants in all possible combinations: “cardiovascular disease,” “cerebrovascular disease,” “CVD,” “novel coronavirus,” “COVID-19,” “SARS-CoV-2,” “cardio-cerebrovascular disease,” “COVID-19.” There were no language restrictions, but the publication time of studies was limited from December 2019 to present. We also manually searched the reference lists of selected articles and related review articles to obtain more useful studies.

### Study Selection

Criteria for inclusion of a study are as follows: 1) patients must be diagnosed with COVID-19; 2) the number of participants in the study must be greater than 20; 3) a comparison of patients with cardio-cerebrovascular disease and patients without cardio-cerebrovascular disease must be included in the study; 4) the study must define the degree of severity of COVID-19 (severe vs. non-severe, or intensive care unit (ICU) vs. non-ICU) and the mortality of COVID-19; 5) the number of COVID-19 with cardio-cerebrovascular disease in severe patients, non-severe patients, deaths and survivors must be reported in the study; and, 6) the study design must be cohort or case-control. Because some studies from MedRxiv and BioRxiv have not been peer-reviewed, we checked whether these studies have finally been peer-reviewed. Those studies that have not been peer-reviewed are indicated in Tables 1 and 2. Two authors independently assessed the studies for eligibility with subsequent consensus through discussion. The disagreement was resolved with involvement of the third author.

**Table 1.** Main Characteristics of Included Studies in Meta-analysis for COVID-19 Severity.

Author, year	Country	Study design	Sample	Gender (M/F)	Median age (years)	Type of CCVD	With CCVD		Without CCVD		Incidence of CCVD
							Mild	Severe	Mild	Severe	
Mendy, A., 2020 (preprint)	USA	Retrospective	689	365/324	49.5 (35.0–64.0)	CaVD	76(22%)	265(78%)	333(95%)	15(5%)	49.49%
Petrilli, C. M., 2020	USA	Prospective	2,729	1,672/1,057	63.0 (51.0–74.0)	CaVD	1,166(61%)	761(39%)	573(71%)	229(29%)	70.61%
Buckner, F. S., 2020	USA	Retrospective	105	53/52	60.0 (23.0–97.0)	CaVD	22(55%)	18(45%)	32(58%)	33(42%)	38.10%
Romero-Sanchez, C.M., 2020	Spain	Retrospective	841	473/368	NR	CeVD	7(50%)	7(50%)	505(61%)	322(39%)	1.66%
Khamis, F., 2020	Oman	Retrospective	63	53/10	NR	CaVD	2(50%)	2(50%)	37(63%)	22(37%)	6.35%
Li, Y., 2020	China	Retrospective	219	89/130	NR	CeVD	2(18%)	9(82%)	125(60%)	83(40%)	5.02%
Xie, Y., 2020	China	Retrospective	62	27/35	63.0 (53.0–73.0)	CaVD	16(48%)	17(52%)	22(76%)	7(24%)	53.23%
Mao, L., 2020	China	Retrospective	214	87/127	NR	CeVD	8(53%)	7(47%)	118(59%)	81(41%)	7.01%
Huang, C., 2020	China	Prospective	41	30/10	49.0 (41.0–58.0)	CaVD	3(50%)	3(50%)	25(71%)	10(29%)	14.63%
Wang, D., 2020 (CaVD)	China	Retrospective	138	75/63	56.0 (42.0–68.0)	CaVD	11(55%)	9(45%)	91(77%)	27(23%)	14.49%
Wang, D., 2020 (CeVD)	China	Retrospective	138	75/63	56.0 (42.0–68.0)	CeVD	1(14%)	6(86%)	101(77%)	30(23%)	5.07%
Guan, W. J., 2020 (CaVD)	China	Retrospective	1,099	640/459	47.0 (35.0–58.0)	CaVD	17(63%)	10(37%)	909(85%)	163(15%)	2.46%
Guan, W. J., 2020 (CeVD)	China	Retrospective	1,099	640/459	47.0 (35.0–58.8)	CeVD	11(73%)	4(27%)	915(84%)	169(16%)	1.36%
Yan, S., 2020 (preprint)	China	Retrospective	168	81/87	51.0 (36.0–62.0)	CaVD	6(50%)	6(50%)	126(81%)	30(19%)	7.14%
Zhang, J. J., 2020	China	Prospective	140	71/69	57.0 (25.0–87.0)	CaVD	5(36%)	9(64%)	77(61%)	49(39%)	10.00%
Zhang, G., 2020(CaVD)	China	Retrospective	221	108/113	55.0(39.0–66.5)	CaVD	9(41%)	13(59%)	157(79%)	42(21%)	9.95%
Zhang, G.,2020(CeVD)	China	Retrospective	221	108/113	55.0(39.0–66.5)	CeVD	4(27%)	11(73%)	162(79%)	44(21%)	6.79%
Cao, M., 2020 (preprint)	China	Retrospective	198	101/97	NR	CaVD	7(58%)	5(42%)	172(92%)	14(8%)	6.06%
Xu, X. W., 2020	China	Retrospective	62	36/26	41.0 (32.0–52.0)	CeVD	0(0%)	1(100%)	29(48%)	32(52%)	1.61%
Wei, Y. Y., 2020	China	Retrospective	167	95/72	NR	CaVD	17(71%)	7(29%)	120(84%)	23(16%)	14.37%

*Note.* CCVD = cardio-cerebrovascular diseases; CaVD = cardiovascular diseases; CeVD = cerebrovascular diseases; NR = not reported.

**Table 2.** Main Characteristics of Included Studies in Meta-Analysis for COVID-19 Mortality.

Author, year	Country	Study design	Sample	Gender (M/F)	Median age (years)	Type of CCVD	With CCVD		Without CCVD		Incidence of CCVD
							Survivor	Death	Survivor	Death	
Mehra, M. R., 2020	USA	Retrospective	8,910	5,518/3,392	NR	CaVD	907(90%)	103(10%)	7,488(95%)	412(5%)	11.34%
Inciardi, R. M., 2020	Italy	Retrospective	99	88/11	67.0 (48.0–86.0)	CaVD	34(64%)	19(36%)	39(85%)	7(15%)	53.54%
Sousa, G. J. B., 2020	Brazil	Retrospective	2,034	1,017/1,053	NR	CaVD	66(43%)	86(57%)	1,837(97%)	45(3%)	7.47%
Soares, R.C. M., 2020	Brazil	Retrospective	1,152	658/494	NR	CaVD	270(51%)	256(49%)	426(68%)	200(32%)	45.66%
Nikpouraghdam, M., 2020	Iran	Retrospective	2,964	1,955/1,099	NR	CaVD	33(89%)	4(11%)	2,692(92%)	235(8%)	1.25%
Halvatsiotis, P., 2020	Greece	Retrospective	90	72/18	65.5 (56.0–73.0)	CaVD	15(80%)	4(20%)	45(67%)	22(33%)	22.09%
Du, R., 2020	China	Prospective	179	97/82	NR	CCVD	17(59%)	12(41%)	141(94%)	9(6%)	16.20%
Yan, Y., 2020	China	retrospective	193	114/79	64.0 (49.0–73.0)	CCVD	4(10%)	35(90%)	81(52%)	73(48%)	20.21%
Wu, C., 2020	China	Retrospective	201	127/74	51.0 (43.0–60.0)	CaVD	4(80%)	16(20%)	141(78%)	40(22%)	9.95%
Ruan, Q., 2020 (CeVD)	China	Retrospective	150	102/48	NR	CeVD	0(0%)	13(100%)	82(60%)	55(40%)	8.67%
Ruan, Q., 2020 (CaVD)	China	Retrospective	150	102/48	NR	CaVD	5(42%)	7(58%)	76(55%)	62(45%)	8.00%
Chen, T., 2020 (CaVD)	China	Retrospective	274	171/103	62.0(44.0–70.0)	CaVD	7(30%)	16(70%)	154(61%)	97(39%)	8.39%
Chen, T., 2020 (CeVD)	China	Retrospective	274	171/103	62.0(44.0–70.0)	CeVD	0(0%)	4(100%)	161(60%)	109(40%)	1.46%
Tu, W. J., 2020	China	Retrospective	174	79/95	NR	CCVD	8(50%)	8(50%)	141(89%)	17(11%)	9.20%
Yuan, M., 2020	China	Retrospective	27	12/15	60.0 (47.0–69.0)	CaVD	0(0%)	3(100%)	17(71%)	7(29%)	11.11%
Zhou, F., 2020	China	Retrospective	191	119/72	56.0 (46.0–67.0)	CaVD	2(13%)	13(87%)	135(77%)	41(23%)	7.85%

*Note.* CCVD = cardio-cerebrovascular diseases; CaVD = cardiovascular diseases; CeVD = cerebrovascular diseases; NR = not reported.

### Data Extraction and Quality Assessment

Two authors independently extracted the data about the baseline characteristics: 1) publication time and location of study; 2) type of study design; 3) sample size; 4) gender distribution; 5) age of patients (range and median age); 6) type of cardio-cerebrovascular disease; 7) number of COVID-19 patients with cardio-cerebrovascular disease and number of patients without cardio-cerebrovascular disease; 8) number of severe COVID-19 patients among patients with the cardio-cerebrovascular disease and patients without cardio-cerebrovascular disease; 9) the number of deaths among patients with cardio-cerebrovascular disease and patients without cardio-cerebrovascular disease. Discrepant judgments of the extracted data were resolved after discussion with the third author.

Two authors independently assessed the quality of each included study using the Newcastle-Ottawa Quality Assessment Scale (NOS; Stang, 2010). The included studies were assessed on 3 aspects: selection, comparability, and outcome/exposure. The scores of each study is detailed in Table 3. Discrepancies in the quality scores were resolved by discussion with the third author.

**Table 3.** The Qualities of Included Studies by Newcastle-Ottawa Quality Assessment Scale.

Study	Selection				Comparability	Outcome/Exposure			Score
	1	2	3	4	1	1	2	3	
Mendy, A.	★		★	★	★★	★		★	7
Petrilli, C. M.	★	★	★		★★	★		★	7
Buckner, F. S.	★	★	★		★★	★		★	7
Romero-Sanchez, C.M.	★	★	★		★★	★		★	7
Khamis, F.	★		★		★★	★		★	6
Li, Y.	★	★	★		★	★	★	★	7
Xie, Y.	★	★	★	★	★★	★	★		8
Mao, L.	★	★	★		★★	★		★	7
Huang, C.	★	★	★	★	★★	★	★		8
Wang, D.	★	★	★		★★	★		★	7
Guan, W. J.	★		★	★	★★	★	★	★	7
Yan, S.	★	★	★		★★	★		★	7
Zhang, J. J.	★	★	★		★★	★		★	7
Zhang, G.	★	★	★	★	★★	★		★	8
Cao, M.	★		★		★★	★		★	6
Xu, X. W.	★	★	★	★	★★	★	★		8
Wei, Y. Y.	★		★		★★	★		★	6
Mehra, M. R.	★		★		★★	★	★	★	7
Inciardi, R. M.	★	★	★	★	★★	★		★	7
Sousa, G. J. B.	★		★	★	★★	★	★	★	8
Soares, R. C. M.	★		★	★	★★	★	★	★	8
Nikpouraghdam, M.	★	★	★		★★	★		★	7
Halvatsiotis, P.	★		★	★	★★	★	★		7
Du, R.	★	★	★	★	★	★		★	7
Yan, Y.	★	★	★		★★	★		★	7
Wu, C.	★	★	★	★	★★	★		★	8
Ruan, Q.	★		★		★★	★		★	6
Chen, T.	★	★	★		★★	★		★	7
Tu, W. J.	★		★		★★	★	★	★	7
Yuan, M.	★	★	★		★	★	★		6
Zhou, F.	★	★	★		★★	★		★	7

### Data Synthesis and Analysis

We used STATA to perform the meta-analysis. Furthermore, the data of each outcome (COVID-19 severity and COVID-19 mortality) were pooled across studies using random-effect (M-H heterogeneity) models separately, to minimize the effects of between-study heterogeneity. The heterogeneity was assessed by χ^2^ Cochran’s Q test and I^2^ statistics, which measures the inconsistency across study results: 0%–25% represents low degree of heterogeneity, 25%–50% represents moderate and 50% or more represents high heterogeneity (Higgins et al., 2003). The fixed-effect model was used when I^2^ < 50%, and the random-effect model was used when I^2^ ≥ 50% (Higgins et al., 2003). We conducted additional subgroup analyses by different types of cardio-cerebrovascular disease (cardiovascular disease, cerebrovascular disease and cardio-cerebrovascular disease) for each outcome. Publication bias was assessed using Begg’s and Egger’s test and visually with funnel plots (Begg & Mazumdar, 1994; Egger et al., 1997). We also performed sensitivity analysis and meta-regression to identify potential sources of heterogeneity.

## Results

### Search Results and Characteristics of the Included Studies

**Figure 1.**
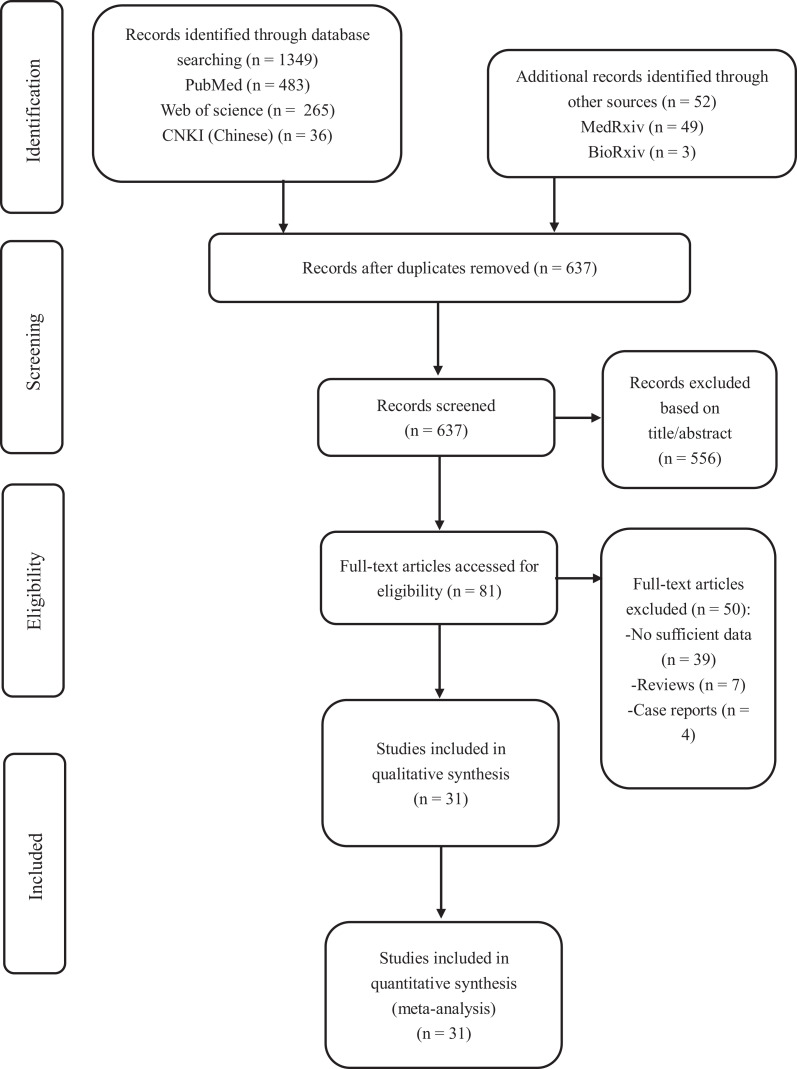
Flow diagram of the study selection and exclusion process.

After initially identifying 1,401 studies from databases including PubMed, Web of Science, CNKI (Chinese), MedRxiv and BioRxiv, we excluded 637 duplicate results. Five hundred and fifty-six studies were excluded by the titles and abstracts. After assessing 81 full-text for eligibility, we excluded 50 full-text studies due to the wrong study design (n = 11) or insufficient data for analysis (n = 39). Thus, 31 studies (27 retrospective studies and four prospective studies) were included in this meta-analysis. Figure 1 displays the process of selection of studies. Three studies had not been peer-reviewed.

There was a total of 23,632 patients, and the characteristics of them are summarized in Table 1 (for disease severity) and Table 2 (for mortality). All selected studies were published in 2020, with sample sizes ranging from 27 to 8,910. The majority of studies were from China (n = 20), four from the USA and seven from other countries (Italy, Brazil, Greece, Iran, Spain and Oman). The median age of patients ranged from 41.0 to 67.0 years, and the prevalence of male gender ranged from 41% to 89%. In the group of COVID-19 severity, the average prevalence of cardio-cerebrovascular disease was 16.27%. In the group of COVID-19 mortality, the average prevalence of cardio-cerebrovascular disease was 15.15%. Seventeen studies (Buckner et al., 2020; Cao et al., 2020; Guan et al., 2020; Huang et al., 2020; Khamis et al., 2020; Y. Li et al., 2020; Mao et al., 2020; Mendy et al., 2020; Petrilli et al., 2020; Romero-Sanchez et al., 2020; Wang et al., 2020; Wei et al., 2020; Xie et al., 2020; Xu et al., 2020; S. Yan et al., 2020; G. Zhang et al., 2020; J. J. Zhang et al., 2020) reported the disease severity of COVID-19 patients with cardio-cerebrovascular disease, and 14 studies (T. Chen et al., 2020; Du et al., 2020; Halvatsiotis et al., 2020; Inciardi et al., 2020; Mehra et al., 2020; Nikpouraghdam et al., 2020; Ruan et al., 2020; Soares et al., 2020; Sousa et al., 2020; Tu et al., 2020; Wu et al., 2020; Y. Yan et al., 2020; Yuan et al., 2020; Zhou et al., 2020) reported the mortality of COVID-19 patients with cardio-cerebrovascular disease.

### Associations Between Cardio-Cerebrovascular Disease and COVID-19

**Figure 2.**
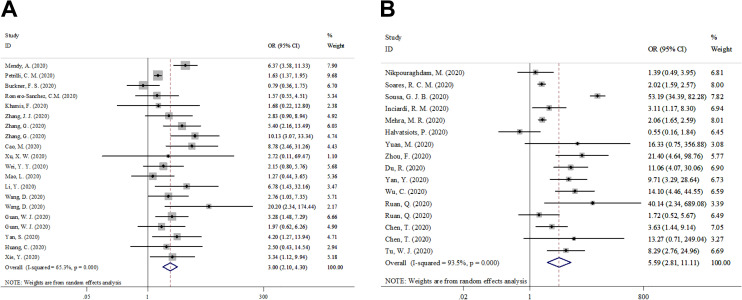
Forest plot for cardio-cerebrovascular disease and COVID-19.

The analysis of the relationship between cardio-cerebrovascular disease and patients with COVID-19 severity included 7,094 participants in 17 studies. The analysis of the relationship between cardio-cerebrovascular disease and patients with COVID-19 mortality included 16,638 participants in 14 studies. Pooled data from the random-effects model showed that the cardio-cerebrovascular disease was associated with increased risk of severe COVID-19 (OR = 3.004; 95% CI = 2.097–4.303; z = 6.00; *p* < 0.001; I^2^ = 65.3%) and death (OR = 5.587; 95% CI = 2.810–11.112; z = 4.91; *p* < 0.001; I^2^ = 93.5%), as shown in Figure 2.

### Publication Bias and Subgroup Analyses

**Figure 3.**
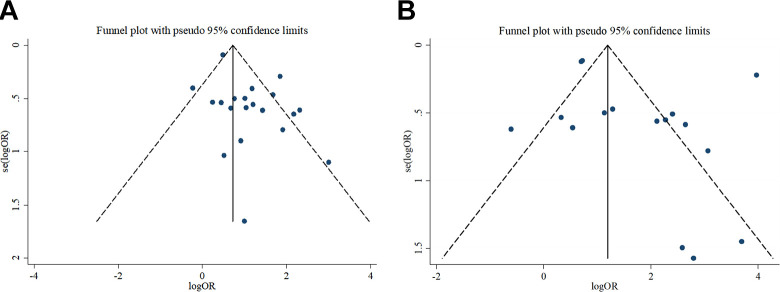
Funnel plot of cardio-cerebrovascular disease and COVID-19.

The funnel-plot analyses with Begg’s and Egger’s test were used to analyze potential publication bias, as shown in Figure 3. However, the funnel-plot for severity was asymmetry, which showed evidence of publication bias for studies reporting on COVID-19 severity and cardio-cerebrovascular disease (Begg’s test: *p* > | z | = 0.469; Egger’s test: *p* > | t | = 0.015) There was no evidence of publication bias for COVID-19 mortality and cardio-cerebrovascular disease (Begg’s test, *p* > | z | = 0.444; Egger’s test, *p* > | t | = 0.216).

**Figure 4.**
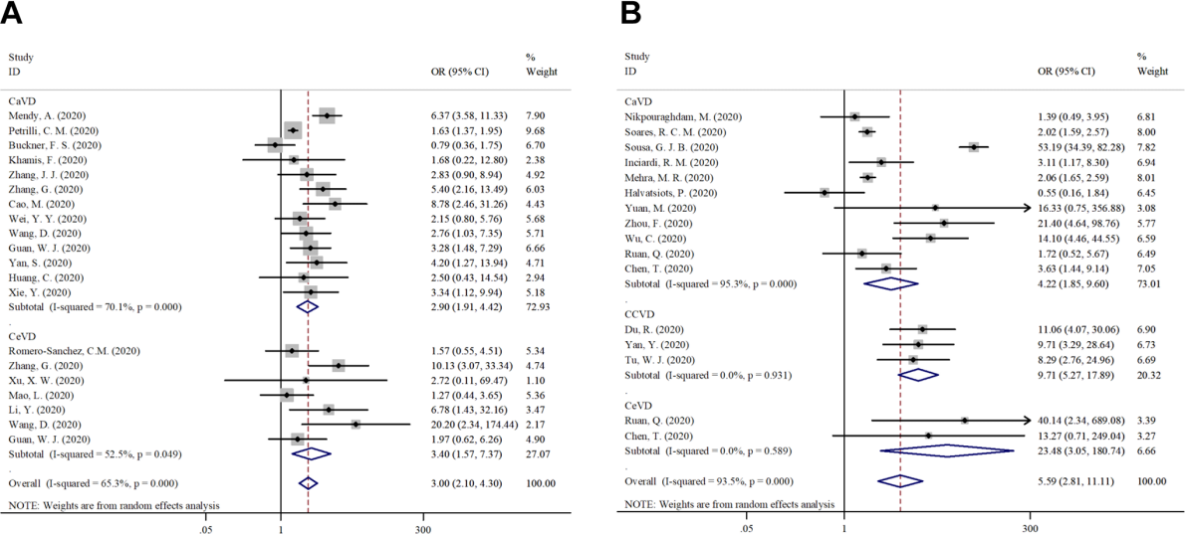
Subgroup analysis by the type of cardio-cerebrovascular disease.

We undertook subgroup analyses by different types of cardio-cerebrovascular disease (cardiovascular disease, cerebrovascular disease and cardio-cerebrovascular disease) for each outcome. It showed similar results to those of the main analysis (Figure 4). The random-effects model showed that cardio-cerebrovascular disease was associated with an increased risk of severe COVID-19 for cardiovascular disease (n = 13, I^2^ = 70.1%; OR = 2.903; 95% CI = 1.906–4.420; z = 4.97; *p* < 0.001) and for cerebrovascular disease (n = 7, I^2^ = 52.5%; OR = 3.400; 95% CI = 1.569–7.368; z = 3.10; *p* = 0.002). It also provided evidence that cardio-cerebrovascular disease was associated with an increased risk of death; for those with cardiovascular disease (n = 11, I^2^ = 95.3%; OR = 4.220; 95% CI = 1.854–9.604; z = 3.43; *p* = 0.001); for those with cerebrovascular disease (n = 2, I^2^ = 0.0%; OR = 23.477; 95% CI = 3.050–180.735; z = 3.03; *p* = 0.002); and for those with cardio-cerebrovascular disease (n = 3, I^2^ = 0.0%; OR = 9.710; 95% CI = 5.271−17.889; z = 7.29; *p* < 0.001.

### Sensitivity Analyses and Meta-Regression

**Figure 5.**
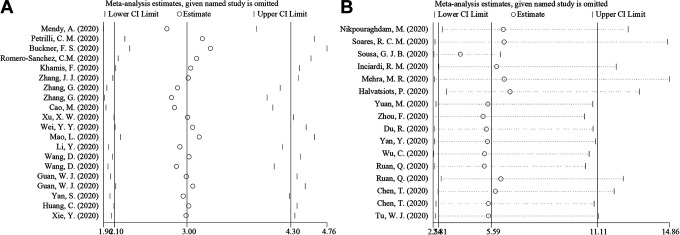
Sensitivity analysis for cardio-cerebrovascular disease and COVID-19.

We conducted a sensitivity analysis by excluding one of the studies and found no significant heterogeneity among included studies for COVID-19 severity (OR = 3.004, 95% CI = 2.097–4.303) and COVID-19 mortality (OR = 5.587, 95% CI = 2.810–11.112), shown in Figure 5.

**Figure 6.**
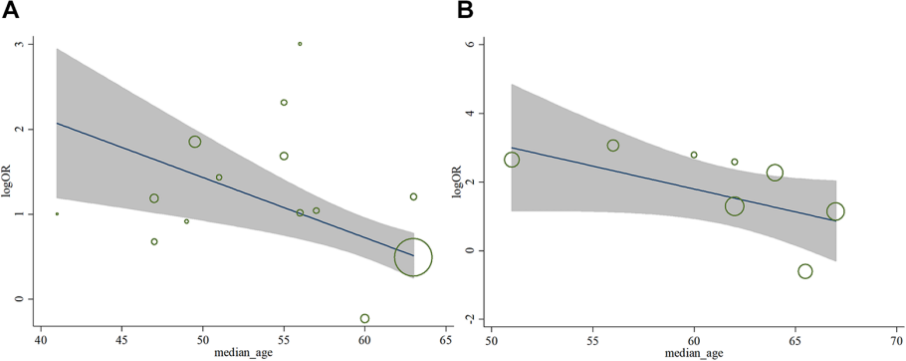
Meta-regression analysis for patients’ median age (years).

Finally, we did meta-regression to evaluate the influence of median age on the heterogeneity among the included studies, and found that the median age was not the source of the heterogeneity (for severity, *p* = 0.181; for mortality, *p* = 0.090), shown in Figure 6.

## Discussion

This systematic meta-analysis demonstrated that COVID-19 patients with cardio-cerebrovascular disease might be at increased risk of developing severe and mortal disease. Patients with cardio-cerebrovascular disease were 3.004 times (95% CI = 2.097–4.303) more likely to be in severe condition than patients without the cardio-cerebrovascular disease and 5.587 times (95% CI = 2.810–11.112) more likely to die than patients without cardio-cerebrovascular disease. Furthermore, based on the results from subgroup analysis, we noticed that those with cerebrovascular disease had greater chance of disease severity and death than those with cardiovascular disease.

Although the exact pathophysiological mechanisms of how cardio-cerebrovascular disease affects COVID-19 patients are still unclear, it can be inferred through some previous studies. First, current studies demonstrated that patients with cardio-cerebrovascular disease were more likely to develop myocardial injury during the course of COVID-19 (T. Chen et al., 2020; Guo et al., 2020). For patients with cardio-cerebrovascular disease, cardiomyocytes could be directly damaged by the SAR-CoV-2 virus, causing systemic inflammatory responses. Furthermore, Huang’s study showed that the imbalanced responses of T helper factor 1 and T helper factor 2 led to a cytokine storm in COVID-19 patients (Huang et al., 2020), resulting in the release of inflammatory post-infection inflammation with a reduction in coronary blood flow, a decrease in the oxygen supply, and the formation of amphibolic coronary plaques and microthrombus. Therefore, patients with cardio-cerebrovascular disease were more likely to develop myocardial injury after SARS-CoV-2 infection, increasing the risk of being critically ill and died. Second, the elevated levels of ACE 2 may be one of the mechanisms by which cardio-cerebrovascular disease affects COVID-19 patients. ACE 2 is a functional receptor for SARS-CoV-2 and has been regarded as the entry point for SARS-CoV-2. Medication that blocks the renin-angiotensin-aldosterone system (RAAS), including angiotensin-converting enzyme inhibitors (ACEI) and angiotensin Ⅱ receptor blockers (ARBs), are commonly used. They increase ACE 2 levels abstractly and lead to more severe diseases in patients (Kow et al., 2020; Mali et al., 2020). However, the role of ACE 2 in coronavirus infection remains controversial and there are insufficient clinical data to prove it (Gross et al., 2020). Third, some of the newly studied drugs for COVID-19 may interact with other cardio-cerebrovascular drugs, which may cause adverse effects and aggravate the patients’ conditions. For example, chloroquine and hydroxychloroquine may interact with antiarrhythmic agents and affect the intracellular pH, which can lead to cardiotoxicity, electrolyte abnormalities, prolonged QT intervals (Driggin et al., 2020; Long et al., 2020). Fourth, due to their low cardiac reserve capacity, once patients with cardio-cerebrovascular disease are infected, the disease is accelerated and lead to poor prognosis (Guo et al., 2020).

This meta-analysis suggested that COVID-19 patients with pre-existing cardio-cerebrovascular disease were associated with poor outcomes. Nurses should be encouraged to assist cardio-cerebrovascular patient to take highly preventive measures. Nurses should not only to pay attention to the occurrence of respiratory dysfunction, but also to pay special attention to signs of cardio-cerebrovascular complications. Meanwhile, the treatment’s safety with ACE inhibitors (ACEI) and angiotensin receptor blockers (ARBs) should be concerned. What’s more, they need to raise awareness of the side effects of various antiviral therapies on cardiovascular-cerebrovascular disease and closely observe the changes in the patient’s condition. Nurses should report the patient’s condition to the clinicians in time so that they can choose correct drugs and formulate the optimal treatment option.

The main limitation of this study is that most of the included studies are from China and there is a lack of COVID-19 patients’ data from other countries. Second, there is a heterogeneity among the included studies. There are 3 articles from MedRxiv that have not been peer-reviewed and we do not rule out the potential bias of them. A larger number of studies are needed to perform more analyses and better confirm our findings.

## Conclusions

In summary, this meta-analysis showed that cardio-cerebrovascular disease had a significant association with the severity and mortality of COVID-19. More careful precautions were suggested for patients with cardio-cerebrovascular. In clinical work, nurses should pay more attention to such patients, so as to better cooperate with doctors and help patients recover.
